# Facile route for preparation of cuprous oxide/copper/cupric oxide nanoparticles by using simultaneous electrochemical and reduction reaction

**DOI:** 10.1016/j.heliyon.2024.e25195

**Published:** 2024-01-30

**Authors:** Ha Xuan Linh, Pham Hoai Linh, Duong Dinh Tuan, Pham Huong Quynh, Nguyen Xuan Hoa, Dang Van Thanh, Hoang Phu Hiep, Nguyen Quoc Dung

**Affiliations:** a International School, Thai Nguyen University, Tan Tinh Ward, Thai Nguyen City, Thai Nguyen, 25000, Viet Nam; b Institute of Materials Science, Vietnam Academy of Science and Technology, Ha Noi, 100000, Viet Nam; c Hanoi University of Industry, 298 Cau Dien Street, Bac Tu Liem District, Hanoi, 100000, Viet Nam; d Faculty of Basic Science, Thai Nguyen University of Medicine and Pharmacy, Luong Ngoc Quyen, Thai Nguyen City, Thai Nguyen, 25000, Viet Nam; e Faculty of Environmental Sciences, University of Science, Vietnam National University, Hanoi, 334 Nguyen Trai Road, Ha Noi, 100000, Viet Nam; f Faculty of Biology, Thai Nguyen Unversity of Education, 20 Luong Ngoc Quyen, Thai Nguyen City, Thai Nguyen, 25000, Viet Nam; g Faculty of Chemistry, Thai Nguyen Unversity of Education, 20 Luong Ngoc Quyen, Thai Nguyen City, Thai Nguyen, 25000, Viet Nam

**Keywords:** Cuprous oxide, Copper, Cupric oxide, Anodic dissolution, Antibacterial

## Abstract

Cuprous oxide/copper/cupric oxide nanoparticles were synthesized through a hybrid process involving anodic dissolution and a controlled redox reaction between NaOH and glucose in the solution. The study demonstrates the structural manipulation of the material by varying the reaction components within the solution. Morphology, structural analyses using SEM (Scanning Electron Microscope), EDX (Energy-dispersive X-ray spectroscopy), TEM (Transmission Electron Microscope), FTIR (Fourier Transform Infrared Spectroscopy), XRD (X-ray diffraction), and XPS (X-ray photoelectron spectroscopy) unveiled the tunability of the material's structure based on the reaction components. Nitrogen adsorption analysis employing the Brunauer-Emmett-Teller (BET) equation confirmed the material's porosity, while Dynamic Light Scattering (DLS) measurements provided insights into the materials' hydrodynamic size and zeta potential. The results demonstrated that by increasing the glucose/NaOH ratio during the reaction, the different structures and morphologies of the distinct products were obtained from the clustering of small nanoparticles to cubic shape and flower-like structure. Antibacterial activity tests conducted on various bacterial strains showed a correlation between the morphology and structure of the material and its antibacterial properties. The highest substantial antibacterial efficacy against all tested bacterial strains at a dosage of 100 μg/L was obtained for the samples with clustering morphology, whereas the remaining materials showed no discernible antibacterial effect against one of the studied bacteria. The results also demonstrated that the sample with a clustering structure exhibited superior antibacterial properties when dispersed in water containing dimethylsulfoxide.

## Highlights

• A new approach to control cuprous oxide/copper/cupric oxide materials.
• Formation materials via a combination of electrolysis and reduction reaction.
• The shape and particle size of a material affect its antibacterial properties.

## 1 Introduction

Metal oxide materials have consistently garnered significant interest among researchers owing to their diverse and intricate structures, which lend themselves to various potential applications. Mainly, when designed at the nano-scale, these materials manifest exceptional characteristics that surpass those of their bulk [[1], [2], [3]]. Among metal oxide materials, those based on copper, with its inherent transition metal nature, multivalence, excellent catalytic activity, abundant reserves, and cost-effectiveness, find a multitude of wide-ranging applications. Copper oxides exist in two primary forms: cupric oxide or copper (II) oxide (CuO), and cuprous oxide or copper(I) oxide (Cu_2_O). Both forms possess a p-type semiconductor structure; however, Cu2O has a larger band gap than CuO [1]. Consequently, their diverse applications can be further enhanced when they coexist within the same material. They have many applications, such as gas sensors [4], electrochemical sensors [5,6], photocatalysts [[7], [8], [9], [10]], supercapacitors [11], antimicrobial activity, antioxidant [12,13], photo-sensing [1] and especially antibacterial activity due to the easy diffusion of Cu^+^ ions into the bacterial membrane [9,14,15]. Therefore, exploring ways to fabricate the nanostructure of Cu and its oxides is a matter of concern. One approach to creating nanostructured CuO involves directly oxidizing copper at elevated temperatures, which can yield CuO in nanowires [16]. However, this reaction necessitates high-temperature conditions and is typically carried out on copper foils, resulting in relatively limited CuO production. By using the hydrothermal method, Cu nanowires can be generated [17], which can subsequently be transformed into various forms, such as CuO nanowires [5] and CuS nanowires [17]. However, it is not feasible to produce bulk quantities through this method. Therefore, it is primarily utilized for applications that require only small quantities, such as sensors [5]. In an effort to achieve mass production, copper oxides are synthesized from readily available copper salts through the solution route [15,[18], [19], [20]]. For example, Wenting Wu et al. [18] fabricated Cu_2_O with different morphologies by slightly changing the reactant using CuSO_4_, NaOH, and glucose. S. Meghana et al. [15] used strong reducing agents hydrazine and NaBH_4_ to reduce Cu^2+^ from the solution. M. Khatami et al. [19] fabricated nano Cu/Cu_2_O precipitated from CuCl_2_ and NaOH solutions. Most methods for preparing copper (I) oxide/copper follow the solution-based pathway using the source of copper (II) salts in an alkaline medium to obtain micro-sized Cu_2_O products. However, the above-mentioned solutions need to be preserved in an acidic environment to avoid hydrolysis, whereas they can be easily polluted by mixed impurities. Therefore, finding a simple method to fabricate a large amount of nanostructured granular Cu_2_O/Cu/CuO materials with high purity remains challenging.

In this investigation, we introduce an innovative fabrication approach utilizing soluble anode electrolysis driven by the intricate interplay of complexation and oxidation reactions, capitalizing on the aldehyde functionality within glucose molecules. Here, glucose serves a dual role: it acts as a complexing agent for the Cu^2+^ ions generated during electrolysis and concurrently functions as a reducing agent for these ions. Consequently, this method offers distinct advantages, including time efficiency, high product purity, and the ability to manipulate diverse morphologies. A key divergence of this technique from the conventional chemical reaction method lies in the continuous generation of Cu^2+^ ions throughout the electrolysis process, facilitating subsequent product formation. In contrast, the conventional chemical process initiates with a pre-established Cu^2+^ concentration that gradually diminishes over the reaction duration, resulting in evolving product properties across the reaction period.

## 2 Materials and methodology

### 2.1 Materials

Pure Cu rod (15 × 0.5 cm, thickness 3.0 mm, 99.95 %), sodium hydroxide [reagent grade, ≥98 %, pellets (anhydrous)], and d (+)-glucose (C_6_H_12_O_6_·H_2_O, for microbiology ≥ 99.8 %) were purchased from Sigma Aldrich. Electrical energy for electrolysis was supplied by a D.C. power source with an applied potential of 50 V. The positive electrode is the copper rod, which is the raw material for making the samples, and the platinum negative electrode acts as the counter electrode. The copper (Cu) and platinum (Pt) electrodes were positioned at a fixed distance of 2 cm from each other within a beaker containing a solution composed of sodium hydroxide (NaOH) and glucose dissolved in deionized water. This entire assembly was then immersed in an Ultrasonic Cleaner with an operational power rating of 80 W.

**Fig. 1:**
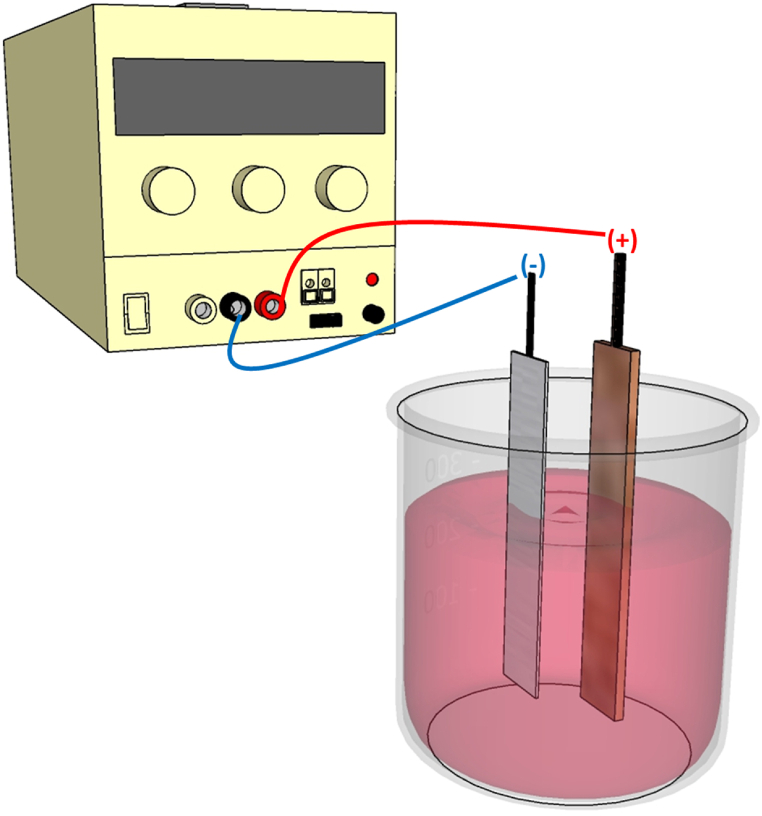
Scheme of the material fabrication process: Cu sheet as an anode and Pt sheet as a cathode.

The formation of the product was observed with the gradual change of the reaction mixture into a red color. After 2 h, vacuum filtration was conducted several times with distilled water until pH = 7. The products were dried in an oven at 80 °C for 8 h. The NaOH concentration was kept at 0.15 M, and the glucose concentration was changed from 0.05, 0.1, 0.15, 0.20, and 0.25 M to evaluate the role of glucose in the formation of the samples. The obtained samples using different glucose concentrations of 0.05, 0.1, 0.15, 0.20, and 0.25 M are denoted as CCN1, CCN2, CCN3, CCN4, and CCN5, respectively. The process for fabricating the material is illustrated in Fig. 1.

### 2.2 Methodology

The morphology, elemental composition and crystalline structure of the as-prepared materials were determined by scanning electron microscopy (SEM, Jeol JSM6510-LV), transmission electron microscopy (TEM), EDX (Energy Dispersive X-ray spectroscope), Fourier Transform Infrared Spectroscopy (FTIR, Nicolet Nexus 670 FTIR) and X-ray diffraction (XRD, Brucker D8 Advance). The hydrodynamic size and zeta potential of CCNn were measured by Dynamic Light Scattering (DLS, NanoPlus, Micromeritics). This measurement facilitates the evaluation of material dispersion and its dynamic stability within an aqueous medium at pH 7 with a sample concentration of approximately 500 ppm. Specific surface area and pore size were studied using nitrogen adsorption at a low temperature (77.35 K) (MicroActive for TriStar II Plus 2.03) based on the Brunauer-Emmett-Teller (BET) equation. The refinement of Rietveld phase composition and phase crystal size in materials is estimated using the Maud program.

The strains used for activity testing were as follows: *Escherichia coli (E. Coli)*, *Staphylococcus aureus (S. aureus)*, *Pseudomonas aeruginosa (P. aeruginosa)*, *Lactobacillus sporogenes (L. sporogenes)*, and *Citrobacter (Citro.)*. The bacteria were cultured in agar trays. Antibacterial ability was tested using the agar well diffusion method. Antibacterial material was dispersed in water containing surfactants (DMSO: Dimethyl sulfoxide 2 %) with 25, 50, and 100 μg/mL concentrations by ultrasonic vibration. Five wells with a diameter of 0.8 cm were made on the agar plate, and 100 μL of the dispersion mixture of the above material was added. The negative control was solvent with a surfactant to disperse the material. The positive control was 30 μL of the antibiotic amoxicillin 50 μg/mL. Antibacterial diameter was determined by the following formula: H = D - d (mm), where D is the diameter of the sterile ring from the center of the hole (mm), and d is the diameter of the agar perforation (mm).

## 3 Results and discussion

**Fig. 2:**
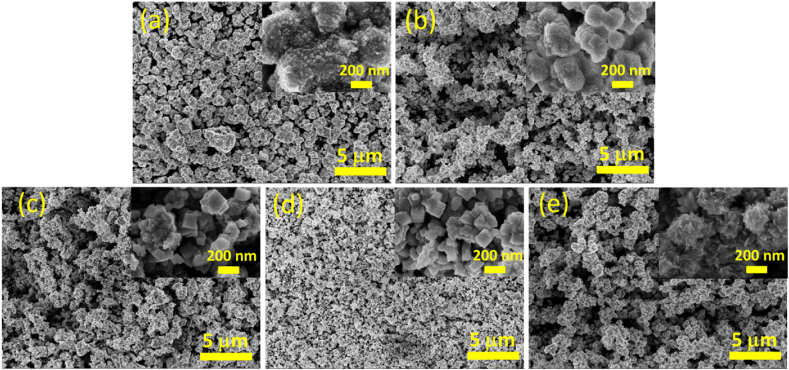
SEM images (a) CCN1, (b) CCN2, (c) CCN3, (d) CCN4, and (e) CCN5.

Fig. 2(a–e) presents the SEM images of the as-prepared samples in particles with fairly uniform sizes. The inset shows that the material exists in different shapes with the change in the synthesis condition. Typically, CCN1 and CCN2 samples exhibit cluster forms with sizes of about 500 nm and 100–200 nm, respectively. Upon examining the insert of Fig. 1, Fig. 2, it is evident that the CCN3 and CCN4 samples exhibit a pronounced cubic morphology, indicating the presence of well-defined crystalline structures. The average particle size of CCN3 and CCN4 are estimated to be approximately 100 nm and 50 nm, respectively. Interestingly, CCN5 has a hierarchical flower-like structure. Zoom in on the SEM images, as depicted in Fig. S1 in the Supplementary Information.

**Fig. 3:**
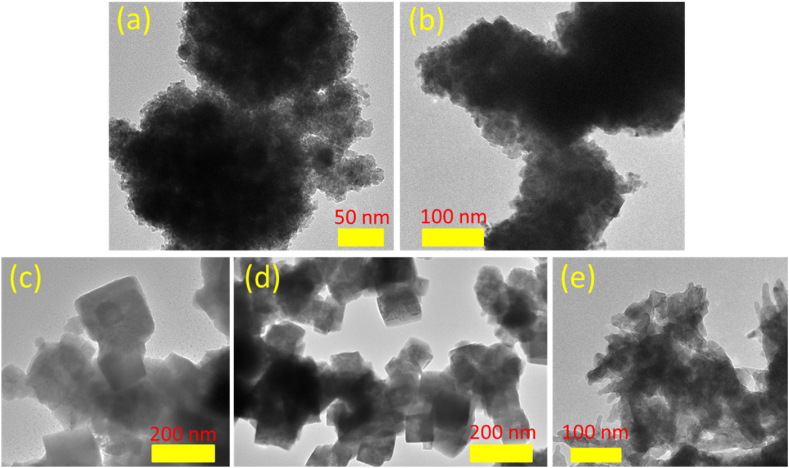
TEM images of CNN (a) CCN1; (b) CCN2; (c) CCN3; (d) CCN4 and (e) CCN5.

The TEM images of the materials presented in Fig. 3 elucidate the particle size characteristics of the material. The TEM images again confirm the modification of the morphology of the samples with a change in reaction parameters. This observation highlights the agglomeration tendencies of particles in CCN1 (Figure [3a]), CCN2 (Figure [3b]), and CCN5 (Figure [3e]), whereas clear cubic crystal particles are evident in CCN3 (Figure [3c]) and CCN4 (Figure [3d]). This can be further elucidated by examining the composition and structure of the material through the characteristic features revealed in Fig. 5, including FTIR, XRD, and EDS. Detailed EDS spectra of materials were performed in Fig. S2.

**Fig. 4:**
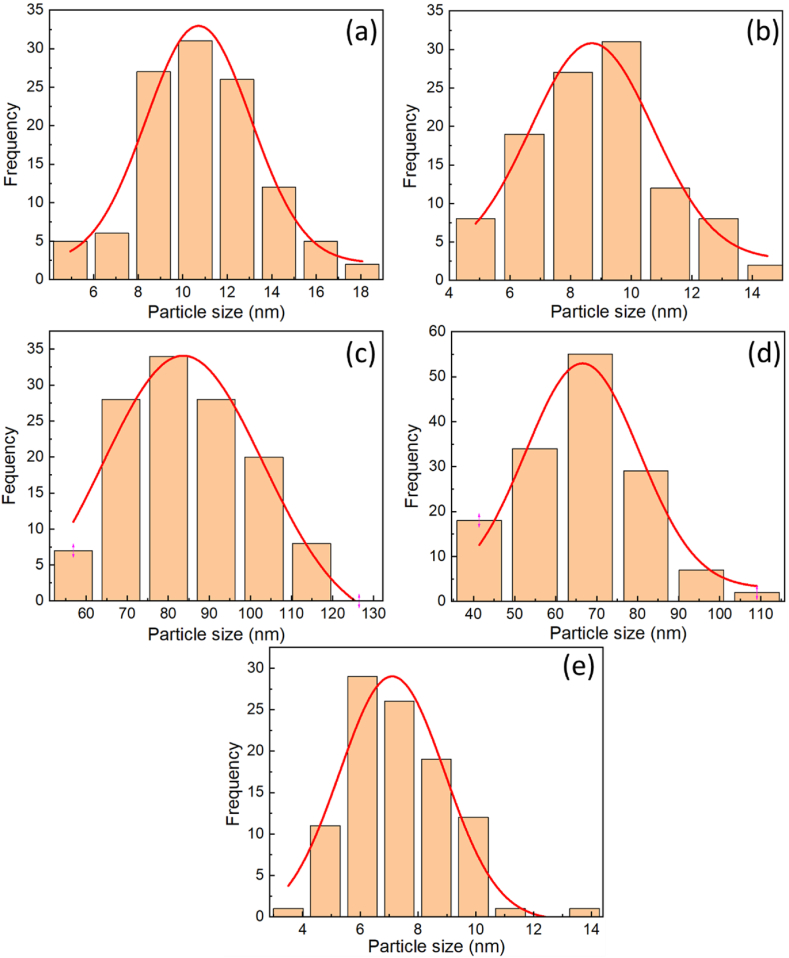
Particle size distribution histogram of (a) CCN1; (b) CCN2; (c) CCN3; (d) CCN4 and (e) CCN5.

**Fig. 5:**
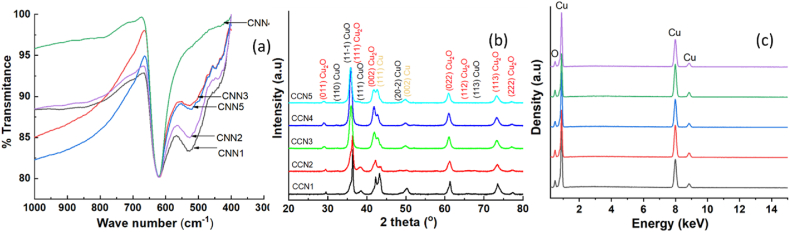
FRIR spectrum (a); XRD pattern (b); and EDS spectrum (c) of CCNn (n = 1–5).

The chemical structure of the synthesized samples was revealed by FTIR spectra, as shown in Fig. 5(a). Though the spectrum is run from 300 to 4000 cm^−1^, the bands from metal oxide are below 1000 cm^−1^. It can be seen that all samples exhibit a strong peak at 621 cm^−1^, attributed to the stretching vibration of Cu–O bonds in Cu_2_O [21]. This confirms the existence of Cu_2_O in the synthesized samples. In addition, the peak around 524 cm^−1^ is attributed to the stretching vibration of the Cu–O bond in CuO NPs also observed in CCN1, CCN2, CCN3, and CCN5 but not detected in CCN4 [22]. This result is completely consistent with the results obtained from XRD, indicating the presence of mixed phases in the prepared system. It can be seen that with a CuO content below 3 %, it is not easy to detect the existence of the CuO phase in the samples by the FTIR spectrum. Although the FTIR peak is basically due to mixed phases, the strong peak at 621 cm^−1^ further affirms that the prepared nanoparticles are predominately Cu_2_O, along with traces of CuO nanoparticles [23]. Additionally, particle size distributions of the materials are graphed based on TEM images, as depicted in Fig. 4. The cluster sizes inclusive of two predominant phases (small Cu_2_O and CuO nanoparticles) are observed at around 11 nm for CCN1 (Figure [4a]) and 9 nm for CCN2 (Figure [4b]). In contrast, a cubic morphology dominated by Cu_2_O is identified, with size of around 80 nm for CCN3 (Figure [4c]) and 70 nm CCN4 (Figure [4d]). Meanwhile CCN5 was characterized by flower-like structures including petals with diameter of about 7 nm (Figure [4e]). Consequently, for an overall assessment of sample sizes, preference was given to size distributions obtained from DLS measurements.

Fig. 5(b) shows the XRD patterns of CCNn materials. The characteristic diffraction peaks of crystalline Cu_2_O (JCPDS No. 77–0199) and metallic Cu (JCPDF No. 04–0836) can be observed in all samples, while the diffraction peaks assigned to the crystalline CuO (JCPDF No. 05–0661) only appear in CCN1-2 samples. Phase composition and crystal size were further confirmed by the Rietveld method using the Maud program [24,25], as presented in Table 1 and Table 2. Cu_2_O is the main component in all CCNn samples, and Cu occupies a smaller amount. CuO is also present in low amounts in CCN1 and CCN2 and is negligible in CCN3, CCN4, and CCN5. Fig. 5(c) displays the EDS spectrum of the material, revealing peaks corresponding to the energy levels of the elements Cu and O without the presence of any other impurities or contaminants. The atomic ratio of Cu to O greater than 2 indicates a significant abundance of Cu_2_O. Further details regarding EDS spectra and the atomic ratio of Cu and O were presented in Fig. S2.

**Table 1:** Phase composition of materials (%) calculated according to the Maud program.

	Cu_2_O (%)	Cu (%)	CuO (%)
CCN1	44.71	38.88	16.41
CCN2	64.07	12.34	23.59
CCN3	71.28	24.58	4.15
CCN4	83.56	13.31	3.14
CCN5	63.64	30.73	5.63

**Table 2:** Calculating crystal size using the Maud program according to the Rietveld method.

No	Materials	Cu_2_O (nm)	Cu (nm)	CuO (nm)
1	CCN1	30.1	10.1	90.1
2	CCN2	15.7	9.9	96.7
3	CCN3	17.8	8.8	97.8
4	CCN4	17.3	19.5	95.3
5	CCN5	17.7	10.2	77.5

**Fig. 6:**
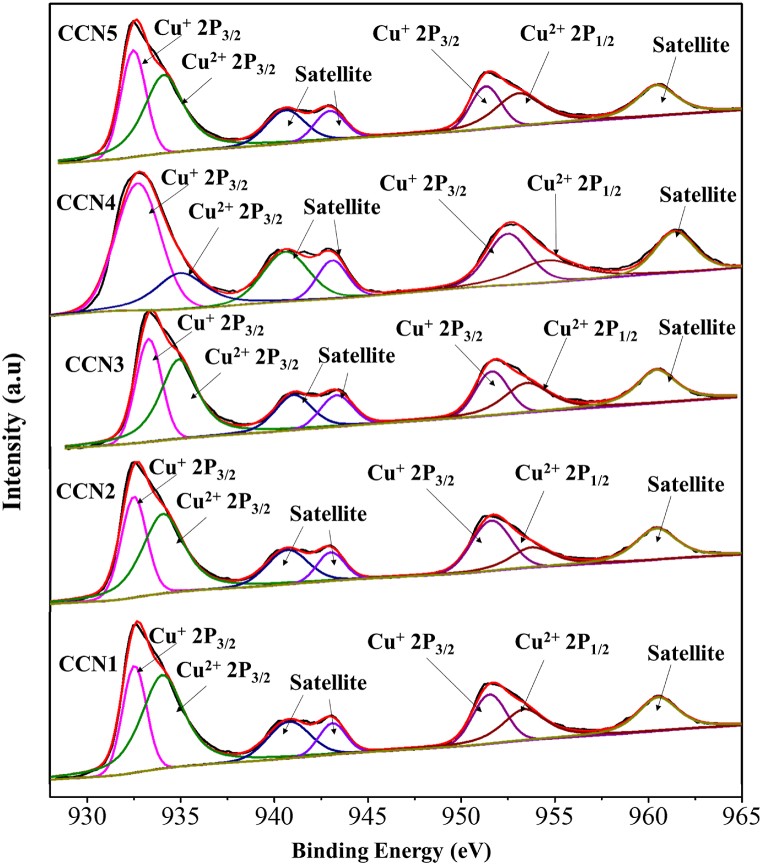
XPS spectrum of CCNn (n = 1–5) for Cu 2p spectrum.

The surface chemistry and elemental composition of CCNn materials were further characterised by XPS analysis. As presented in Fig. S3, the long-range XPS spectrum of all CCNn materials reveals the presence of Cu and O elements only, suggesting the high-purity formation of copper oxide in CCNn materials. On the other hand, Fig. 6 exhibits the Cu 2p core-level spectrum of all CCNn materials, which can be deconvoluted into numerous peaks. Specifically, in the Cu 2p_3/2_ and Cu 2p_1/2_ binding energy regions, the peaks at 932.2 eV and 952.7 eV could be assigned to Cu^+^, whereas the peaks located at 934.8 eV and 954.6 eV could be ascribed to Cu^2+^; besides, three fitting peaks at 941.2 eV, 942.8 eV, and 962.2 eV could be attributed to satellite peaks. The presence of these Cu species validates the formation of Cu_2_O and CuO on the surface of CCNn materials, which are in agreement with XRD results. One can note that from XRD analysis, metallic Cu could be observed, but no exact Cu phase could be noticed in XPS spectra. As reported in the literature [26,27], the binding energies of Cu and Cu^+^ are almost identical; thus, it is difficult to differentiate the phase of Cu and Cu^+^ from Cu 2p XPS spectra. Moreover, the contents of Cu^+^ and Cu^2+^ in different CCNn materials were also measured. Typically, CCN1 and CCN2 samples contain higher CuO content than others; meanwhile, CCN3, CCN4, and CCN5 possess more Cu_2_O in their structure. This result is consistent with the XRD analysis results discussed earlier. On the other hand, the O 1s core-level spectrum of all CCNn materials can also be deconvoluted into two main peaks, as shown in Fig. S4, and the peaks at 529.5 eV, 531 eV, and 532.16 eV could be observed, corresponding to Cu–O bond, lattice oxygen of Cu2O and surface-absorbed oxygen species [28].

With Cu_2_O being the predominant component, we propose the reaction mechanism as follows:

The Cu rod acts as a positive electrode, a source of Cu^2+^, as shown by Eq (1)

(1) Cu ⟶ Cu^2+^ + 2e

In an alkaline medium, Cu^2+^ is hydroxidized by Eq (2):

(2) Cu^2+^ + 2OH^−^ ⟶ Cu(OH)_2_

Cu(OH)_2_ forms a complex with glucose:
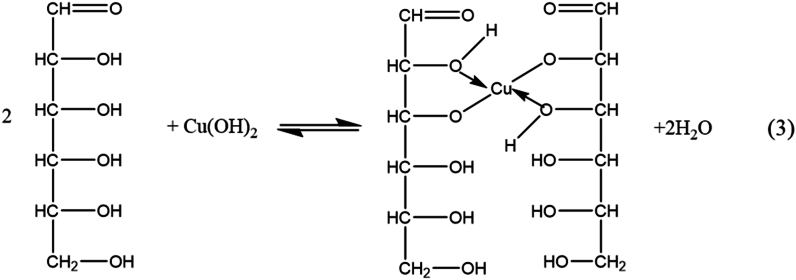


Due to the equilibrium depicted above, the predominant form of copper hydroxide exists in a complex form.

Under ultrasonic waves, a redox reaction occurs:
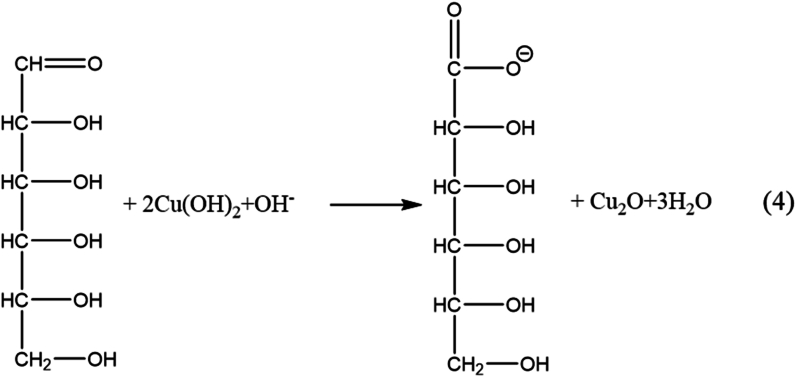


With the above process, with the strong chelating role of glucose with Cu(OH)_2_ and under the influence of ultrasonic energy. Furthermore, ultrasound induces crystal nucleation to occur in a way that favors the development of more particles rather than the growth of individual ones, which may be the reason for the formation of smaller-sized particles.

The appearance of minority products of CuO and Cu are side reactions that can be explained as part of Cu^2+^ in a complex form with glucose being reduced at the cathode (Pt electrode), as shown in Eq (5).
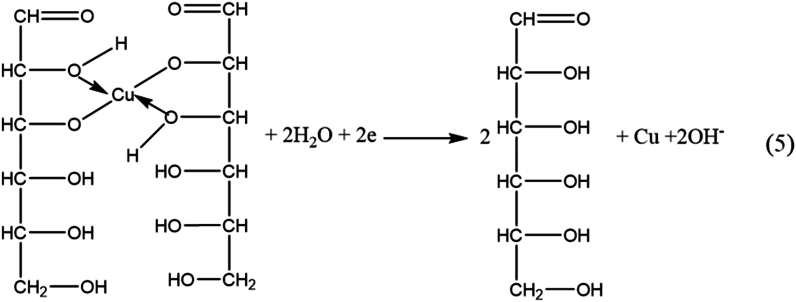


and a part of CuO produced by the pyrolysis of Cu(OH)_2_ by Eq. (6).

(6) Cu(OH)_2_ ⟶ CuO + H_2_O

When the glucose concentration in the reaction increased, most of Cu^2+^ was formed in a complex form with glucose, so the amount of Cu^2+^ in the form of Cu(OH)_2_ was very small. Therefore, from CCN3 to CCN5, the characteristic diffraction peak of CuO disappeared.

The hierarchical flower-like structure of CCN5 can be explained by the strong complexation of Cu^2+^ with glucose; due to a large excess of glucose, the crystal nuclei of Cu_2_O did not grow but agglomerated to form this structure. Thus, materials with suitable shapes and sizes can be obtained by controlling the proper ratio between glucose and NaOH. It can be seen that glucose not only acts as a complexing agent with Cu^2+^ to prevent the formation of Cu(OH)_2_ precipitates but also as a reducing agent for Cu(II) to Cu(I), contributing to the generation of Cu_2_O (Eq [3,4]). The presence of glucose combined with ultrasonic vibration allows the creation of various shapes and sizes of the products, which strongly affect their antibacterial properties. Furthermore, the Cu rod undergoes rapid electrolysis induced by the current, combined with a reaction occurring at high concentrations of glucose and NaOH in the solution. As a result, this method enables a mass production capability of up to several grams in a single manufacturing cycle.

**Fig. 7:**
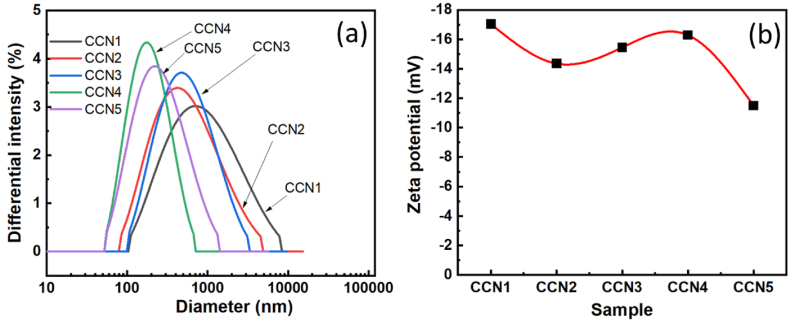
(a) Laser distribution and (b) zeta potential of CCNn samples.

**Fig. 8:**
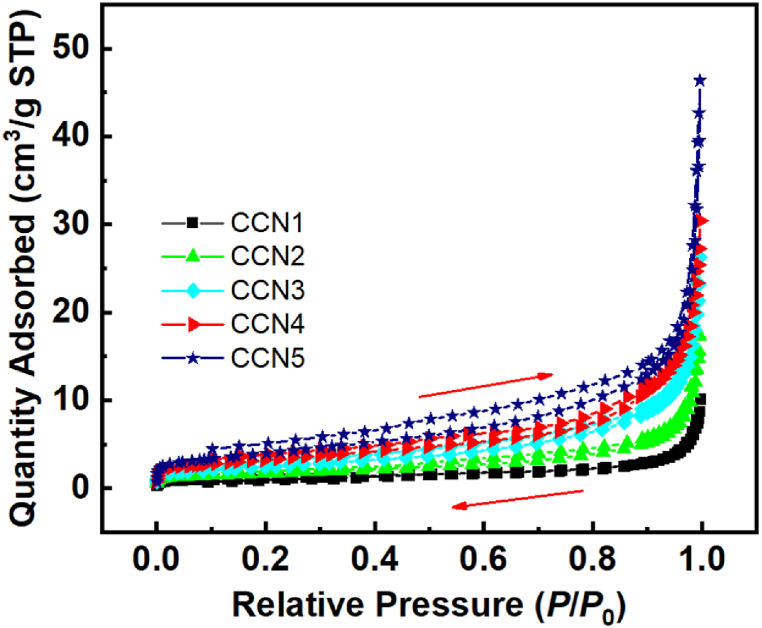
N_2_ – adsorption/desorption isotherms of CCNn samples.

The size distribution potential and zeta potential of the materials are presented in Fig. 7. As presented in Fig. 7(a), the laser distribution gradually decreased in CCN1 to CCN2, slightly increased in CCN3, and decreased again in CCN4 before rising comparatively in CCN5. This finding is relatively consistent with the SEM images in Fig. 2. Nevertheless, the size of the laser distribution is larger than that observed from the SEM images, which could be attributed to the agglomeration of particles. In CCN1, CCN2, and CCN4, the size of the laser distribution is wider, validating that many small particles are agglomerated together; meanwhile, particles in CCN3 and CCN4 with cubic-form show less agglomeration, resulting in narrower laser distribution. Fig. 7(b) illustrates the zeta potential results of all materials. Our research results are quite similar to those on copper and copper oxides previously published [[29], [30], [31], [32], [33], [34], [35], [36], [37]] (Table 3). Fig. 8 shows the nitrogen adsorption–desorption curves for all samples. The curves are of type IV and have H3 hysteresis loops for all samples corresponding to parallel plate-shaped pores. Table 4 shows the comparison of porosity characteristics with previous reports [36,[38], [39], [40], [41]]. The as-prepared materials in the present study possess relatively larger specific surface areas than those in previous works. The large specific surface areas and appropriate pores contribute to increased contact between the material and bacterial cells, potentially enhancing antibacterial efficiency. In addition, the ability to release Cu ions easily also helps increase antibacterial effectiveness.

**Table 3:** Comparison of the zeta potential of the material with previous reports.

Materials	Zeta (mV)	Particle size (nm)	Ref
Cu_2_O	−14.7	400	[29]
Cuboctahedral Cu_2_O	+15.4	310	[30]
Cu_2_O nanoparticles	Around from −14.0 to −26.2	2–50 nm	[31]
Cu_2_O nanocubes	+0.27	192	[32]
Cu_2_O octahedra	+6.9	511	
Cu_2_O rhombic dodecahedra	+14.5	373	
Cu	Around −20	104.6	[33]
Cu	−22.3	Around 20 (by Shearer fomular from XRD pattern)	[35]
cCuO	−5.6	17.8	[34]
wCuO	−22.3	24	
aCuO	−16.5	–	
eCuO	−15.6	–	
Cu/Cu_2_O composite	+24		[36]
CuO nanoparticles	−21	167.1	[37]
CCN1	−17.04	∼500	This work
CCN2	−14.35	100–200	
CCN3	−15.45	∼100	
CCN4	−16.29	∼50	
CCN5	−11.50	∼200

**Table 4:** Comparison of the porosity characteristics between CCNn and other materials.

Materials	BET specific surface area (m^2^/g)	Pore volume (cm^3^/g)	Pore size (nm)	Ref
CeO_2_–CuO/Cu_2_O/Cu	4.92			[38]
Cu_2_O NPs	17.0	0.04615	10.80	[39]
CuO/Cu_2_O composite hollow sphere	17.1	0.04	3.4	[40]
Cu_2_O/CuO	10.392	0.04552	17.430	[41]
Cu_2_O/Cu	2.5369	0.0036	5.6938	
Cu_2_O/Cu	3.041			[36]
CCN1	4.1842	0.01178	11.25	This work
CCN2	5.9325	0.02122	14.30	
CCN3	11.2345	0.03697	13.12	
CCN4	17.7210	0.05120	14.93	
CCN5	8.4335	0.03115	14.78	

The aforementioned results demonstrate that the material exhibits the necessary properties to be considered for antibacterial applications.

**Fig. 9:**
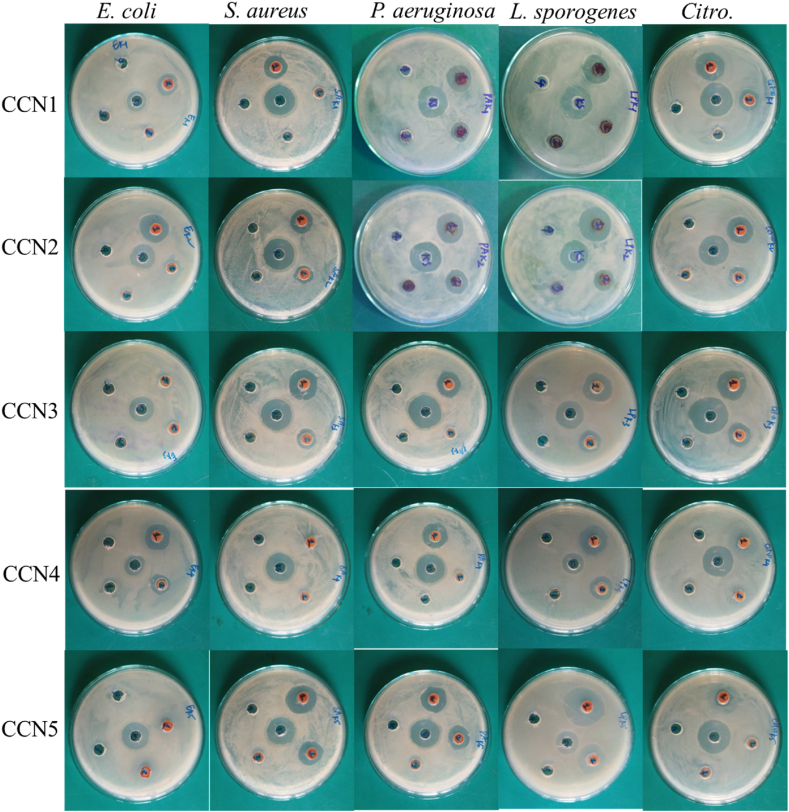
Antibacterial imaging of materials by agar well diffusion method.

As shown in Fig. 9 and Tables S1–S5 (shown in Supplementary Information), the antibacterial ability of the obtained materials was indicated by the diameter of the antibacterial ring. At the CCNn concentration of 25 μg/mL, the material had no activity against the tested bacteria. At 50 μg/L, the antibacterial activity of nanoparticles appeared in some strains. At 100 μg/L, all samples were resistant to bacterial strains. In particular, CCN1 and CCN2 exhibited antibacterial activity against all five strains of bacteria studied. The different antibacterial results show the influence of different structures and particle sizes. The underlying mechanism responsible for the antibacterial activity of the material can be elucidated as follows:

Gram-negative bacteria have a thin peptidoglycan layer between the cytoplasmic and outer membranes. By contrast, Gram-positive bacteria lack the outer membrane but have a thick peptidoglycan layer. Differences in membrane structure affect the antibacterial properties of the materials [42]. Three mechanisms have been summarized: oxidative stress, dissolved metal ions, and non-oxidative mechanism [15], in which oxidative stress and dissolved metal ions are predominant. In the oxidative stress mechanism, reactive oxygen species (ROS) (such as O_2_^−^, ·OH, and H_2_O_2_) can induce oxidative stress and accelerate the decline and death of bacteria. In the mechanism of dissolved metal ions, metal ions are slowly released from metal oxide. They are absorbed through the cell membrane, followed by direct interaction with the functional groups of proteins and nucleic acids, such as mercapto (–SH), amino (–NH), and carboxyl (–COOH) groups, thereby damaging the enzyme activity, changing the cell structure, affecting the normal physiological processes, and ultimately inhibiting the growth of the microorganism. Copper ions released from CCNn can implement the dissolved metal ions mechanism, and CCNn can generate ROS that implements the oxidative stress mechanism. Due to the abovementioned factors, copper oxide nanoparticles show different antibacterial activities against various bacterial strains. Table 5 shows the relatively good antibacterial ability of the CCNn materials by agar well diffusion [19,[42], [43], [44], [45], [46], [47]]. According to observations, at a concentration of 100 μg/L, CCN1 and CCN2 demonstrated antibacterial effectiveness against all tested strains, while CCN3, CCN4, and CCN5 showed no activity against any of the strains. This could be attributed to a higher CuO ratio and smaller size observed in CCN1 and CCN2, as observed via TEM (Fig. 3) and depicted in the Particle Size Distribution Histogram (Fig. 4). This comparative outcome aids in selecting the optimal material for various purposes.

**Table 5:** Comparison of antibacterial properties of materials by agar well diffusion method with previous reports.

Materials	Bateria	Dose (μg/mL)	Antibacterial diameter	Ref
CuO NPs	*S. aureus*	100	22.5	[43]
	*S. epidermis*	100	21.6	
	*S. pyogens*	100	22.3	
	*E. coli*	100	20.3	
	*S. marcescom*	100	20.0	
	*K. pneumonia*	100	18.6	
CuO NPs	*E. coli*	1000	26	[44]
	*S. aureus*	–	nonsensitive	
Cu_2_O	*S. aureus*	2000	15.5	[42]
	*P. aeruginosa*	500	15.2	
	*E. coli*		completely inhibit	
Cu_2_O NPs	*S. aureus*	20	20.2	[45]
	*C. alibicans*	20	19.5	
Cu_2_O NPs	*P.aeruginosa*	100	18	[46]
	*E. coli*	100	23	
	*S. aureus*	300	20	
Cu_2_O nanocubes	*B. thuringiensis*	2000	24	[47]
	*P. aeruginosa*	2000	23	
Cu/Cu_2_O NPs	*P.aeruginosa*	100	12	[19]
Cu_2_O/Cu/CuO	*E. Coli (−)*	100	21	This work
	*S. aureus (+)*	100	17	
	*P.aeruginosa (−)*	100	21	
	*L. sporogenes (+)*	100	25	
	*Citro (−)*	100	20	

## 4 Conclusion

Cuprous oxide/copper/cupric oxide (Cu_2_O/Cu/CuO) nanoparticles were successfully prepared by a combination of anodic dissolution and controlled redox reaction between NaOH and glucose in the solution. The as-prepared materials exhibited distinct morphologies by altering glucose concentrations with a fixed NaOH amount (i.e., 0.15 M). At low glucose concentrations (i.e., 0.05 and 0.1 M), the materials had particle-clustered shapes with Cu and CuO phases. Once the concentration of glucose increased to 0.15 and 0.2 M, cubic-shaped materials were obtained. When the glucose was further increased to 0.25 M, a flower-like structure was formed. Accordingly, the synthesized materials with different sizes and shapes exhibited different antibacterial properties. CCN1 and CCN2 samples showed the highest antibacterial capability against all five strains of bacteria tested, whereas CCN3 to CCN5 exhibited less efficiency. This work provides useful information about constructing material structures with controllable shapes and sizes and the effect of structure on antibacterial activity.

## CRediT authorship contribution statement

**Ha Xuan Linh:** Writing – original draft, Formal analysis, Conceptualization. **Pham Hoai Linh:** Formal analysis, Conceptualization. **Duong Dinh Tuan:** Formal analysis, Data curation. **Pham Huong Quynh:** Data curation. **Nguyen Xuan Hoa:** Data curation. **Dang Van Thanh:** Data curation. **Hoang Phu Hiep:** Data curation. **Nguyen Quoc Dung:** Writing – review & editing, Formal analysis, Conceptualization.

## Declaration of competing interest

The authors declare that they have no known competing financial interests or personal relationships that could have appeared to influence the work reported in this paper.
