# Supplementary material for: Facile route for preparation of cuprous oxide/copper/cupric oxide nanoparticles by using simultaneous electrochemical and reduction reaction
Source: Heliyon. 2024 Jan 30;10(3):e25195. doi: 10.1016/j.heliyon.2024.e25195 (PMC10862522; doi:10.1016/j.heliyon.2024.e25195)
Supplement: Multimedia component 1 [file mmc1.docx]

**Supplementary Information**

**Facile route for preparation of cuprous oxide/copper/cupric oxide nanoparticles by using simultaneous electrochemical and reduction reaction**

Ha Xuan Linh^a^, Pham Hoai Linh^b^, Duong Dinh Tuan^a^, Pham Huong Quynh^c^, Nguyen Xuan Hoa^d^, Dang Van Thanh^d,e^, Hoang Phu Hiep^f^, Nguyen Quoc Dung^g*^

*^a^International School, Thai Nguyen University, Tan Tinh Ward, Thai Nguyen City, Thai Nguyen 25000, Vietnam*

*^b^Institute of Materials Science, Vietnam Academy of Science and Technology, Ha Noi, Viet Nam*

*^c^Hanoi University of Industry, 298 Cau Dien Street, Bac Tu Liem District, Hanoi, Vietnam*

*^d^Faculty of Basic Science, Thai Nguyen University of Medicine and Pharmacy, Luong Ngoc Quyen, Thai Nguyen 25000, Vietnam*

*^e^Faculty of Environmental Sciences, University of Science, Vietnam National University, Hanoi, 334 Nguyen Trai Road, Ha Noi City 100000, Vietnam*

*^e^Faculty of Biology, Thai Nguyen Unversity of Education, 20 Luong Ngoc Quyen, Thai Nguyen City, Thai Nguyen 25000, Vietnamg*

*^g^Faculty of Chemistry, 20 Luong Ngoc Quyen, Thai Nguyen City, Thai Nguyen 25000, Vietnam*

Corresponding author.

*E-mail address*: [dungnq@tnue.edu.vn](mailto:dungnq@tnue.edu.vn) (Nguyen Quoc Dung)

**3. Results and discussion**

**
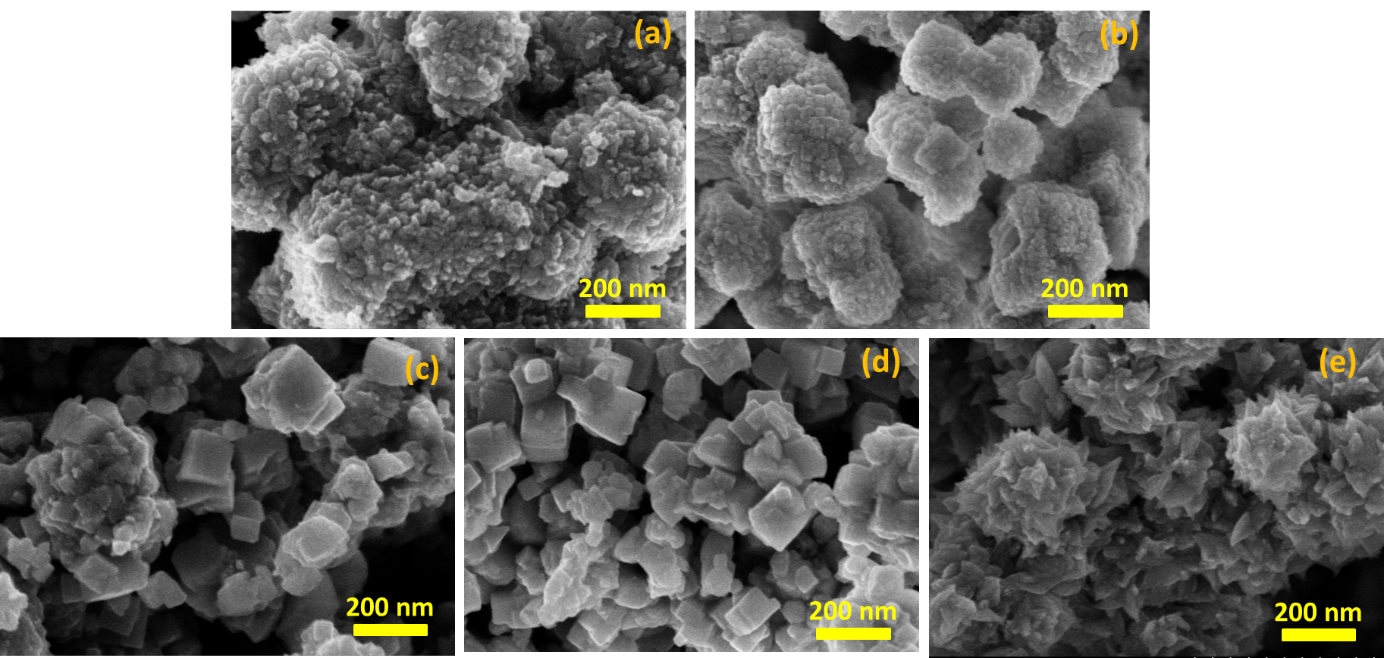
**

Figure S1. FE-SEM images of (a) CCN1; (b) CCN2; (c) CCN3; (d) CCN4; and (e) CCN5


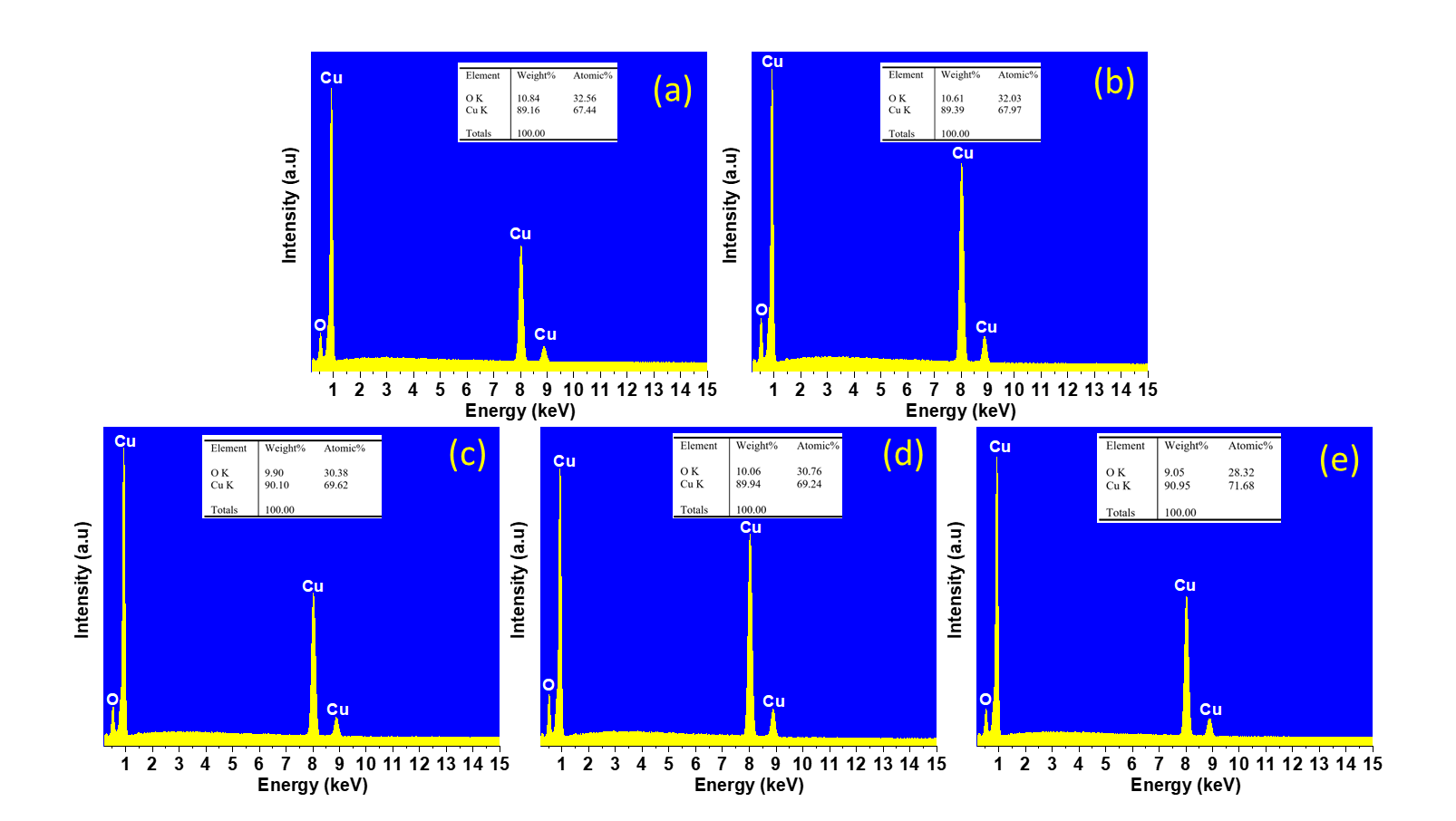


Figure S2. EDS spectra of (a) CCN1; (b) CCN2; (c) CCN3; (d) CCN4 and (5) CCN5


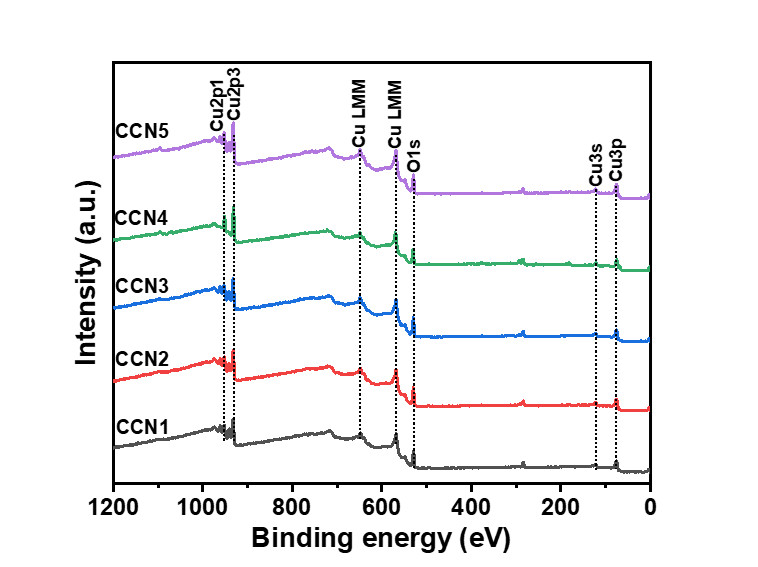


Figure S3. Long-range XPS spectrum of CCNn materials


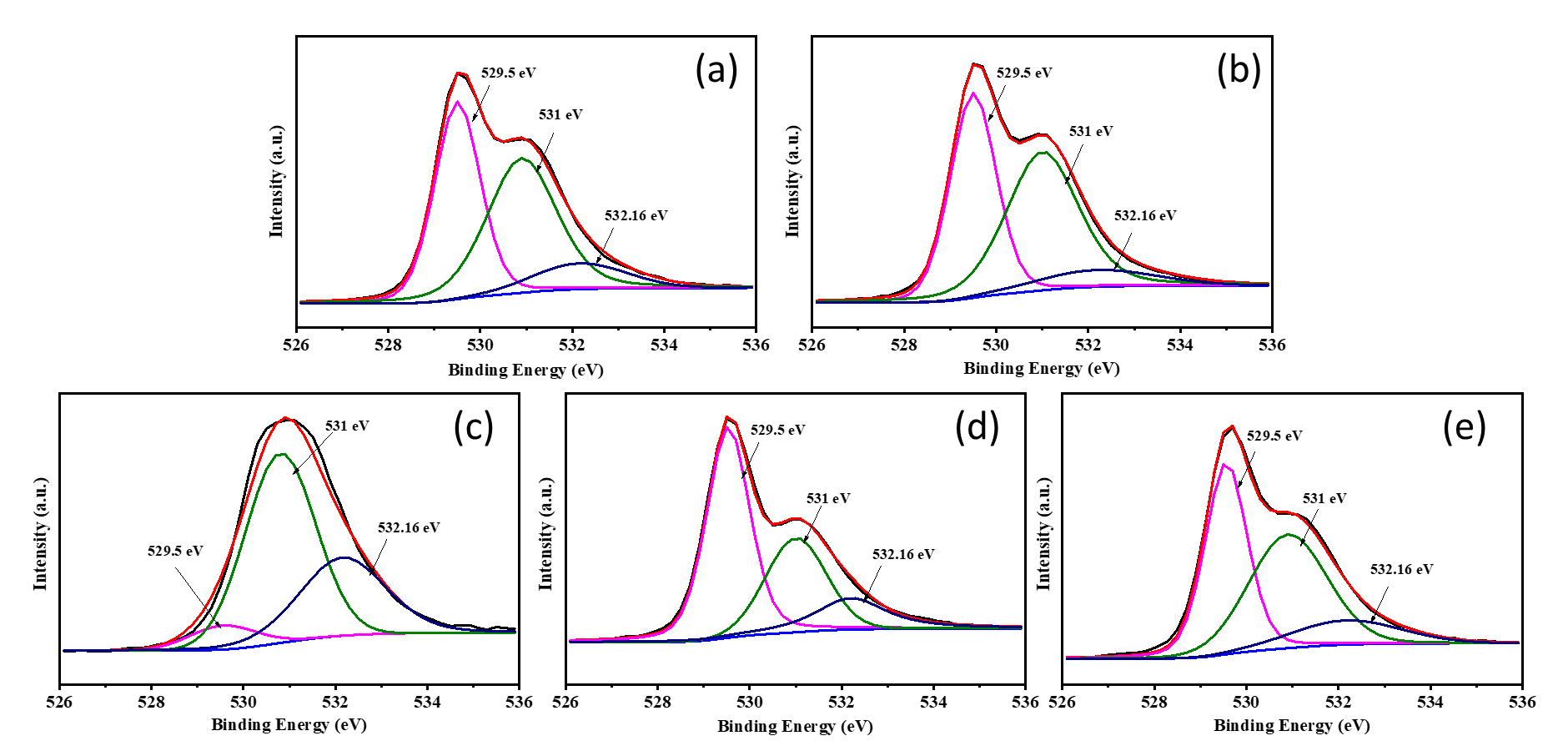


Figure S4. XPS spectrum of CCNn (n=1-5) for O 2p spectrum

Table S1. Antibacterial activity of CCN1 and control samples against different bacterial strains

| Samples | Dose (µg/L) | *E. Coli* | *S. aureus* | *P.aeruginosa* | *L. sporogenes* | *Citro* |
| --- | --- | --- | --- | --- | --- | --- |
|  |  | Antibacterial diameter (mm) | | | | |
| CCN1 | 100 | 16 | 17 | 18 | 17 | 20 |
| CCN1 | 50 | 0 | 0 | 15 | 0 | 15 |
| CCN1 | 25 | 0 | 0 | 0 | 0 | 0 |
| H_2_O | 0 | 0 | 0 | 0 | 0 | 0 |
| Amoxi. | 50 | 15 | 22 | 22 | 16 | 22 |

Table S2. Antibacterial activity of CCN2 and control samples against different bacterial strains

| Samples | Dose (µg/L) | *E. Coli* | *S. aureus* | *P.aeruginosa* | *L. sporogenes* | *Citro* |
| --- | --- | --- | --- | --- | --- | --- |
|  |  | Antibacterial diameter (mm) | | | | |
| CCN2 | 100 | 21 | 17 | 21 | 25 | 20 |
| CCN2 | 50 | 0 | 10 | 16 | 14 | 14 |
| CCN2 | 25 | 0 | 0 | 0 | 0 | 0 |
| H_2_O | 0 | 0 | 0 | 0 | 0 | 0 |
| Amoxi. | 50 | 15 | 21 | 22 | 15 | 22 |

Table S3. Antibacterial activity of CCN3 and control samples against different bacterial strains

| Samp. | Dose (µg/L) | *E. Coli* | *S. aureus* | *P.aeruginosa* | *L. sporogenes* | *Citro* |
| --- | --- | --- | --- | --- | --- | --- |
|  |  | Antibacterial diameter (mm) | | | | |
| CCN4 | 100 | 19 | 0 | 15 | 20 | 0 |
| CCN4 | 50 | 13 | 0 | 0 | 17 | 0 |
| CCN4 | 25 | 0 | 0 | 0 | 0 | 0 |
| H_2_O | 50 | 0 | 0 | 0 | 0 | 0 |
| Amoxi. |  | 14 | 22 | 22 | 15 | 20 |

Table S4. Antibacterial activity of CCN4 and control samples against different bacterial strains

| Samp. | Dose (µg/L) | *E. Coli* | *S. aureus* | *P.aeruginosa* | *L. sporogenes* | *Citro* |
| --- | --- | --- | --- | --- | --- | --- |
|  |  | Antibacterial diameter (mm) | | | | |
| CCN3 | 100 | 0 | 21 | 20 | 20 | 21 |
| CCN3 | 50 | 0 | 14 | 0 | 15 | 16 |
| CCN3 | 25 | 0 | 0 | 0 | 0 | 0 |
| H_2_O | 50 | 0 | 0 | 0 | 0 | 0 |
| Amoxi. |  | 15 | 21 | 22 | 20 | 23 |

Table S5. Antibacterial activity of CCN3 and control samples against different bacterial strains

| Samp. | Dose (µg/L) | *E. Coli* | *S. aureus* | *P.aeruginosa* | *L. sporogenes* | *Citro* |
| --- | --- | --- | --- | --- | --- | --- |
|  |  | Antibacterial diameter (mm) | | | | |
| CCN5 | 100 | 0 | 21 | 20 | 30 | 10 |
| CCN5 | 50 | 0 | 18 | 17 | 21 | 0 |
| CCN5 | 25 | 0 | 0 | 0 | 0 | 0 |
| H_2_O | 50 | 0 | 0 | 0 | 0 | 0 |
| Amoxi. |  | 14 | 22 | 22 | 15 | 20 |
